# Is Zolpidem Associated with Increased Risk of Fractures in the Elderly with Sleep Disorders? A Nationwide Case Cross-Over Study in Taiwan

**DOI:** 10.1371/journal.pone.0146030

**Published:** 2015-12-30

**Authors:** Yih-Jing Tang, Shinn-Ying Ho, Fang-Ying Chu, Hung-An Chen, Yun-Ju Yin, Hua-Chin Lee, William Cheng-Chung Chu, Hui-Wen Yeh, Wei-Shan Chiang, Chia-Lun Yeh, Hui-Ling Huang, Nian-Sheng Tzeng

**Affiliations:** 1 Department of Family Medicine, Center for Geriatrics and Gerontology, Taichung Veterans General Hospital, Taichung, Taiwan; 2 School of Medicine, Chung Shan Medical University, Taichung, Taiwan; 3 Institute of Bioinformatics and Systems Biology, National Chiao Tung University, Hsinchu, Taiwan; 4 Department of Biological Science and Technology, National Chiao Tung University, Hsinchu, Taiwan; 5 Department of Computer Science, Tunghai University, Taichung, Taiwan; 6 Department of Nursing, Tri-Service General Hospital, School of Nursing, National Defense Medical Center, Taipei, Taiwan; 7 Institute and Department of Mathematics, National Tamkang University, New Taipei City, Taiwan; 8 Department of Psychiatry, Tri-Service General Hospital, School of Medicine, National Defense Medical Center, Taipei, Taiwan; 9 Student Counseling Center, National Defense Medical Center, Taipei, Taiwan; McLean Hospital/ Harvard Medical School, UNITED STATES

## Abstract

**Background:**

We conducted a study using a case-crossover design to clarify the risk of acute effects of zolpidem and benzodiazepine on all-sites of fractures in the elderly.

**Design of study:**

Case-crossover design.

**Methods and Materials:**

Elderly enrollees (n = 6010) in Taiwan’s National Health Insurance Research Database with zolpidem or benzodiazepine use were analyzed for the risk of developing fractures.

**Results:**

After adjusting for medications such as antipsychotics, antidepressants, and diuretics, or comorbidities such as hypertension, osteoarthritis, osteoporosis, rheumatoid arthritis and depression, neither zolpidem nor benzodiazepine was found to be associated with increased risk in all-sites fractures. Subjects without depression were found to have an increased risk of fractures. Diazepam is the only benzodiazepine with increased risk of fractures after adjusting for medications and comorbidities. Hip and spine were particular sites for increased fracture risk, but following adjustment for comorbidities, the associations were found to be insignificant.

**Conclusion:**

Neither zolpidem nor benzodiazepine was associated with increased risk of all-site fractures in this case cross-over study after adjusting for medications or comorbidities in elderly individuals with insomnia. Clinicians should balance the benefits and risks for prescribing zolpidem or benzodiazepine in the elderly accordingly.

## Introduction

Fractures are a significant and growing health problem for elderly individual, and are associated with increased morbidity and mortality [1–3]. Though the absolute incidence of fractures in the elderly has decreased in recent years, the resulting economic burden and mortality have increased [1, 4, 5]. In France, a 5-year (2000–2004) analysis of 2,625,743 death certificates showed a 2.2% incidence of fractures, resulting in a crude number of deaths associated with fractures of 57,753, while the number associated with osteoporotic fractures was 46,849 (1.85% and 1.78% of all deaths, respectively) [2]. Fractures also impose high medical costs during and even after surgical treatment; in the United States, costs for pelvic, hip and tibia fractures accrue in the second year following injury ($5,121, $3,930, and $3,828, respectively) [6].

Previous studies have shown that hypertension [7], osteoarthritis and osteoporosis [8], diabetes mellitus, depression, hyperlipidemias, heart failure, dementia and cardiovascular disease [9–11] are associated with increased risk of fractures in the elderly. Vitamin D deficiency is also a known risk factor for fractures [12, 13]. Aging and the ensuing reduction in physical activity, could also contribute to fractures [13]. Falling injuries may contribute to pelvic [14, 15], hip [16] and other fractures in the elderly [17].

In previous studies, zolpidem [18–21] and benzodiazepine (BDZ) [22, 23] were reported to be associated with increased risk of elderly hip or non-vertebral fractures. Few studies examined the association between hypnotics and non-hip fractures. Furthermore, a case-crossover study design might be more suitable to clarify the acute or short-term effects on risk in all-sites fractures in the elderly associated with zolpidem [24, 25], since the hangover effects or complex sleep behaviors associated with these drugs (e.g., zolpidem-related somnambulism) usually do not persist for more than a day [26–30].

This study used the Taiwan National Health Insurance Research Database (NHIRD) to determine the association of zolpidem or BZDs and all-sites of fractures in the elderly from 2000 to 2010. As of June 2009, Taiwan’s National Health Insurance (NHI) Program covered 22,928,190 people, exceeding 99% of the population. The NHI also has contracts with 97% of medical providers in Taiwan [31]. The NHI uses diagnostic coding in accordance with the International Classification of Diseases, 9th Revision, Clinical Modification (ICD-9-CM) diagnostic criteria [32]. Each fracture diagnosis is made by board-certified orthopedic surgeons. The Bureau of National Health Insurance randomly reviews the charts of 1 per 100 ambulatory and 1 per 20 in-patient claim cases to verify diagnosis accuracy [33].

## Materials and Methods

### Data Sources

This research was based on a population-base cohort study. The data were taken from the Longitudinal Health Insurance Database (LHID) for 2005 published by the NHI Bureau. Taiwan’s NHI was launched in March 1995 [34] and covered from over 25 million Taiwanese. It also represented approximately 98% of the Taiwanese [35] who lived in Taiwan for more than four months.

The longitudinal database is a subset of the NHIDatabase consisting of 1 million samples from Taiwanese covered by the NHI in 2005 [36]. It contains patient medical records for in-patient, out-patient, and ambulatory care. Each sample is enrolled in the LHID with codes for diagnoses, surgical operations, dental services, prescription drugs, medical institutions, physicians, and registration data, all based on the International Classification of Disease, 9th revision, Clinical Modification (ICD-9-CM) [37].

### Study Subjects

The present study tracked patients over the age of 65 who were using zolpidem or BDZs. In addition, the study also discussed patients diagnosed with sleep disorders (ICD-9-CM codes 307.41–307.49 and 780.50–780.89) and their impact on fractures. The index date was defined as the date on which subjects were diagnosed with fractures by ICD-9-CM codes for fractures of the hip (820), humerus (812), forearm (813), wrist (814), spine (805, 806), and any other sites (800–829). Patients who had been previously diagnosed with fractures before the index date were excluded. For patients, who had suffered a stroke and were paralyzed for a period of time, it was impossible to measure the extent of paralysis and the recovery time. Therefore, we excluded subjects with a stroke diagnosis. Regarding diseases, such as dizziness and locomotor problems, it could not be confirmed whether the patient was paralyzed or not. Consequently, we did not take these diseases into account. All study subjects had used hypnotics within six months prior to the index date.

### Data Collection of Study Subject Characteristics

**Fig 1:**
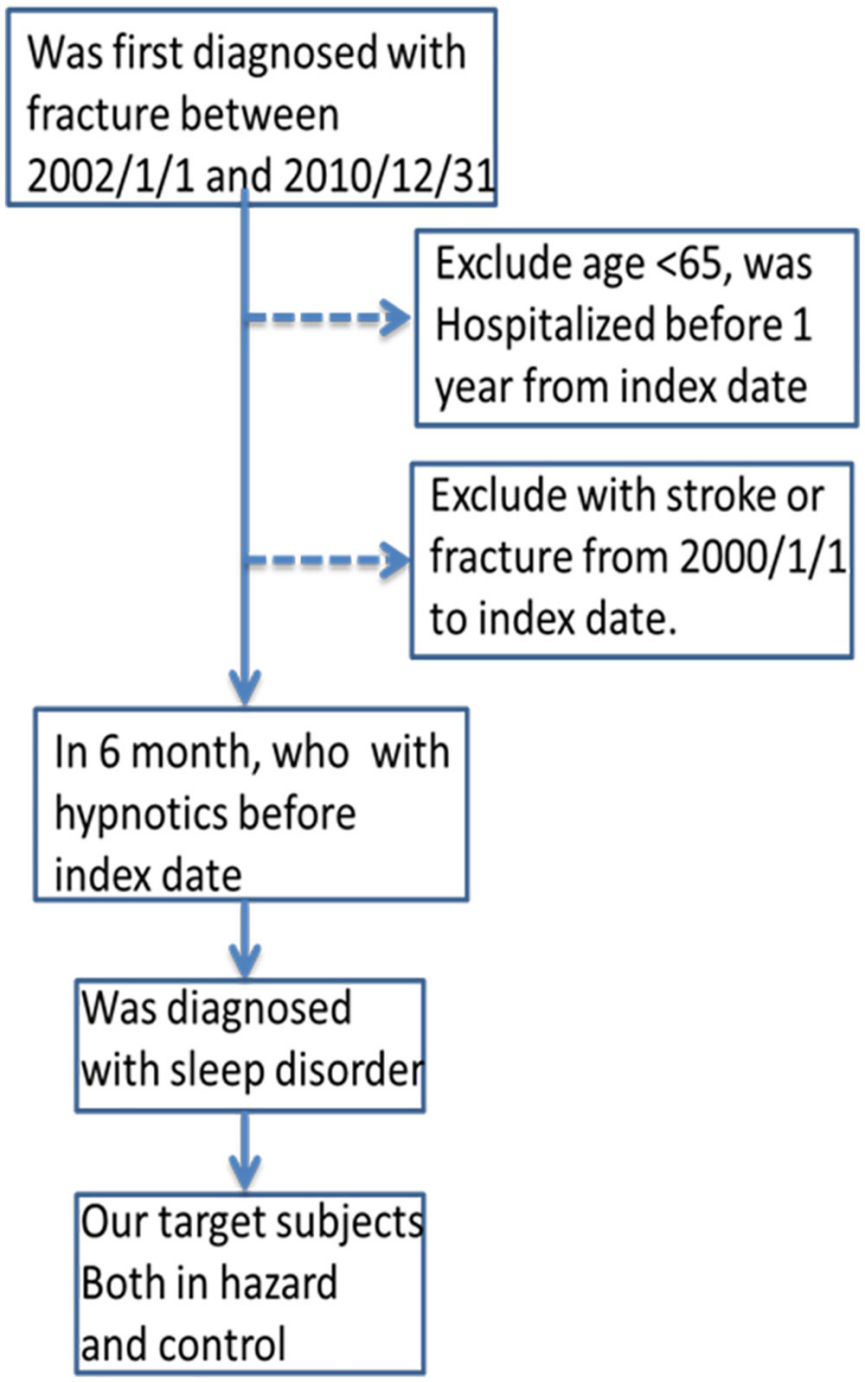
Flowchart for the selection of the sample from the National Health Insurance Research Dataset.

Comorbidities or coexisting medications that might interact with the risk of fractures were adjusted for. The confounding factors with potential to change between case and control groups were adjusted in our analysis based on Chen, et al. [38]. These confounding factors included comorbidities and coexisting medications and were identified by outpatient data. In this study, comorbidities were defined as experiencing the following medical conditions within one year prior to the index date in each control and hazard period: Hypertension, osteoarthritis, osteoporosis, diabetes mellitus, dementia, rheumatoid arthritis, heart failure, Parkinson’s disease, non-stroke cerebrovascular diseases, chronic obstructive pulmonary disease (COPD), benign prostate hyperplasia (BPH), hyperlipidemias, gout, hypothyroidism, anemia, and depression. Medications including antidepressants, diuretics and antipsychotics were also adjusted for. Fig 1 shows the data collection process; 5 weeks was defined as a washout period and 1 day was used to observe if the patient took the drug or not. We had 4 controls matched with 1 case; therefore, in total, we needed 6 months to ensure that the data were included.

### Ethical Statement

The protocol for this study conformed to the Helsinki Declaration, and was approved by the Institutional Review Board of Cathay General Hospital (permission code: CGH-P101089). Personal identifiers from the database were encrypted or otherwise stripped prior to analysis, and the need for written informed consent was waived by the Institutional Review Board.

### Case-crossover Design

**Fig 2:**
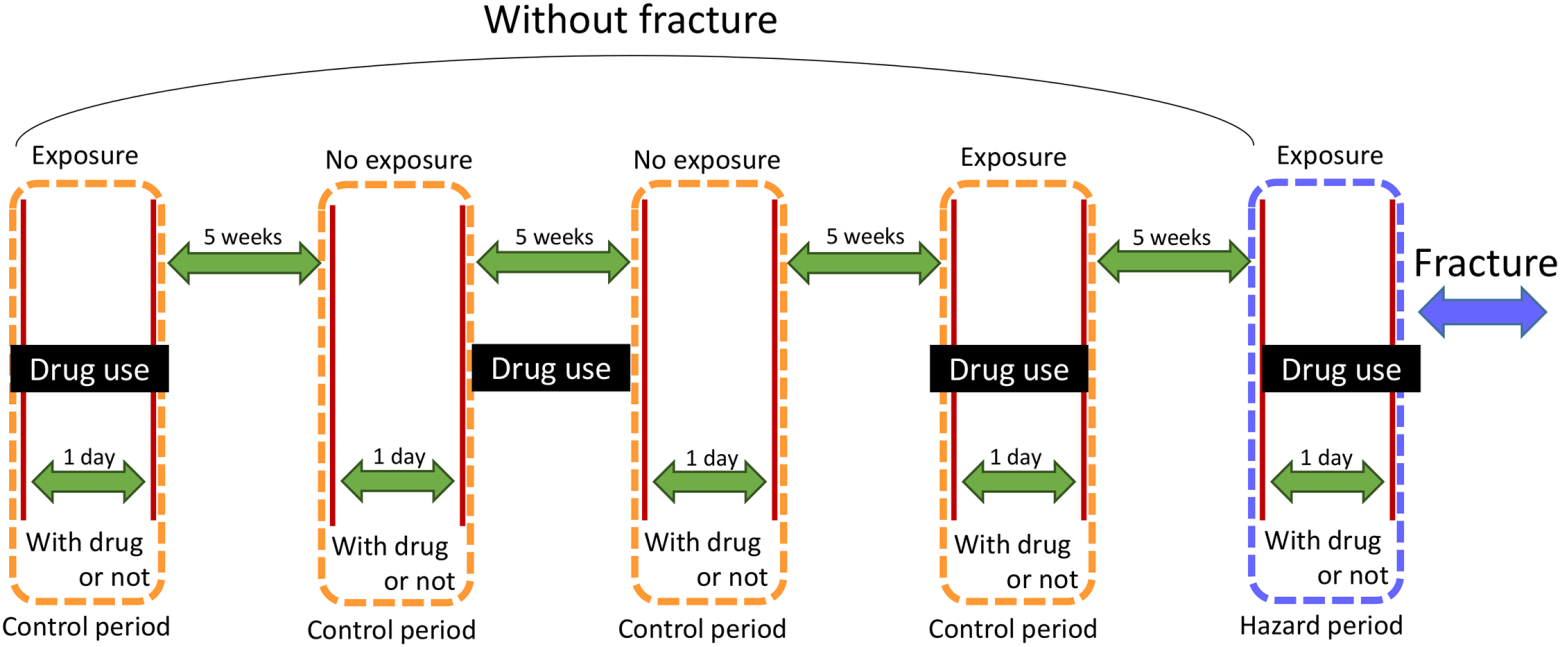
The study design of case cross-over: 1) Hazard period: patients who had been diagnosed with fractures, 2) Control period: patients who had not been diagnosed with fractures, 3) Washout period: 5 weeks for the washing out of residues of drug reaction.

The case-crossover design is widely used to study the acute effects of drug over short durations [29, 30]. Each enrolled subject has used the drug at least once in the past. The case-crossover design was compared against a control of their own data prior to fracture diagnosis. This study enrolled samples between 2002 and 2010 with washout periods of 5, 10, 15, and 20 weeks; thus each study subject had 4 controls. In each control group, we only observed 1 day whether the patient took drug before the 5-week-washout period. The control time windows are 1 day same as the case time window (Fig 2). The impact of BDZ and zolpidem lasts less than 1 day; thus zolpidem or BZD use was defined as use 1 day prior to the fracture. A case-crossover study design can avoid confounding factors unrelated to time such as gender, but cannot avoid the factors that are related to time (e.g., comorbidity and other medication).

### Statistical Analyses

Statistical Product and Service Solution (SPSS) v19.0 is widely used for analytical computing, data mining, and predictions. The present study used conditional logistic regression analyses odds ratio (OR) and adjusted OR. Within 95% confidence interval p < 0.05 was the level of significance for fracture. Stratified analysis was used to predict changes to risk of fracture under different conditions of BDZ and zolpidem use.

## Results

Table 1 shows that more women have sleep disorders in this cohort (n = 4071, 67.7%) than men (n = 1939, 32.3%). Most patients with sleep disorders were grouped in ages 65–74 (n = 2482, 40.7%) and 75–84 (n = 2809, 46.8%). In our data, most patients with sleep disorders also had hypertension (n = 3726, 62.0%) and osteoarthritis (n = 3226, 53.7%) and some had exhibited chronic renal failure (n = 359, 5.9%) and rheumatoid arthritis (n = 176, 2.9%) in the past year.

**Table 1:** Baseline characteristics of study patients (n = 6010).

Characteristics	n (%)
Gender	
Female	4071(67.7%)
Male	1939(32.3%)
Age	
65–74	2482(40.7%)
75–84	2809(46.8%)
> = 85	759(12.6%)
Comorbidity	
Hypertension	3726(62.0%)
Osteoarthritis	3226(53.7%)
Osteoporosis	937(15.6%)
Diabetes mellitus	1522(25.3%)
Anemia	524(8.7%)
Depression	583(9.7%)
Dementia	325(5.4%)
Rheumatoid arthritis	176(2.9%)
Heart Failure	570(9.5%)
Parkinson Disease	318(5.4%)
non-stroke CVD	385(6.4%)
COPD	563(9.4%)
CRF	353(5.9%)
BPH	831(13.8%)
hyperlipidemia	1419(23.6%)
Gout	734(12.2%)

CVD = cerebral vascular disease, CRF = chronic renal failure, BPH = benign prostate hypertrophy,

Table 2 shows the ORs in patients with sleep disorders. In the hazard period, more patients used BDZ (n = 1155, 19.2%) than used zolpidem (487, 8.1%). Use of BDZ (4399, 18.0%) was also more frequent than that of zolpidem (1755, 7.3%) in the control periods. The crude OR of fractures in patients using BDZ was 1.17 (CI: 1.06–1.30, p<0.05). After adjusting for use of antidepressants, antipsychotics and diuretics, the OR was decreased to 1.13 (1.02–1.26, p < 0.05). For zolpidem, the adjusted odds ratio was 1.23 (CI: 1.06–1.44, p < 0.05). After adjusting for comorbidities, used of either BDZ (OR = 1.08, CI: 0.97–1.22, p = 0.14) or zolpidem (OR = 1.13, CI: 0.96–1.34, p = 0.14) was not significantly associated with fractures (Table 2).

**Table 2:** Risk of fractures due to the use of hypnotics in elderly insomnia patients.

Hypnotics	No. of case	No. of control	Crude OR	*Adjusted OR	**Adjusted OR
	(n = 6010)	(n = 24040)	(95% CI)	(95% CI)	(95% CI)
Benzodiazepine	1155(19.2%)	4339(18.0%)	1.17(1.06–1.30) ^†^	1.13(1.02–1.26) ^†^	1.08(0.97–1.22)p = 0.142
Zolpidem	487(8.1%)	1755(7.3%)	1.27(1.09–1.48) ^†^	1.23(1.06–1.44) ^†^	1.13(0.96–1.34)p = 0.136

OR, odds ratio: CI, 95% confidence interval.

*Adjusted for the use of antidepressant, antipsychotics and diuretics.

**Adjusted for hypertension, osteoarthritis, osteoporosis, rheumatoid arthritis, and depression.

^†^p < 0.05 means “indicates significance for the comparison between patients who were and were not using drugs”.

Table 3 shows stratified analysis results revealing different conditions that could impact the likelihood of fractures. In this study, subjects aged 65–74 with BZD use were associated with increased risk for fractures, as OR = 1.36 (CI: 1.24–1.49, p < 0.001), but, after for adjusting comorbidities, the OR was not significant at 1.11 (CI: 0.93–1.33, p = 0.24). Using conditional logistics regression analysis, patients in different age groups had no association with increased risk for fractures, regardless of BDZ or zolpidem use. Before adjustment, both women (OR = 1.30, CI: 1.20–1.41, p < 0.001) and men (OR = 1.32, CI: 1.17–1.49, p < 0.001) using BDZ had significantly elevated risk of fractures. However, after adjusting for comorbidities, neither women nor men showed significantly increased fracture risk. In patients without depression, the OR was 1.17 (CI: 1.03–1.31, p < 0.05) with fractures after BDZ use.

**Table 3:** Risk of fractures due to the use of hypnotics in elderly patients stratified by age group and gender.

		Benzodiazepin			Zolpidem		
Characteristics	n	n (%)	Crude OR(95%CI)	Adjusted OR(95%CI)	n (%)	Crude OR(95%CI)	Adjusted OR(95%CI)
Age							
65–74	12210	2061(33.1%)	1.36(1.24–1.49)^‡^	1.11(0.93–1.33)p = 0.238	788(12.6%)	1.27(1.08–1.48)^†^	1.16(0.89–1.52)p = 0.274
75–84	14045	2769(39.3%)	1.27(1.14–1.43)^‡^	1.08(0.92–1.26)p = 0.374	1177(16.7%)	1.44(1.20–1.73)^†^	1.16(0.92–1.45)p = 0.227
> = 85	3795	664(18.3%)	1.13(0.90–1.41)p = 0.29	1.05(0.77–1.44)p = 0.747	277(7.3%)	1.32(0.91–1.93)p = 0.148	0.98(0.61–1.58)p = 0.919
Gender							
Female	20355	3760(18.5%)	1.30(1.20–1.41)^‡^	1.07(0.94–1.23)p = 0.320	1488(7.3%)	1.31(1.14–1.50)^‡^	1.18(0.96–1.45)p = 0.108
Male	9695	1734(17.9%)	1.32(1.17–1.49)^‡^	1.12(0.92–1.37)p = 0.249	754(7.8%)	1.41(1.15–1.73)^†^	1.05(0.79–1.39)p = 0.793
Hypertension							
Yes	17668	3582(20.3%)	1.17(0.98–1.27)p = 0.092	1.10(0.96–1.26)p = 0.186	1599(9.1%)	1.26(1.05–1.51)^†^	1.17(0.96–1.41)p = 0.121
No	17382	1912(15.4%)	1.05(0.94–1.18)p = 0.385	1.07(0.88–1.30)p = 0.503	643(5.2%)	1.17(0.87–1.56)p = 0.305	1.05(0.76–1.45)p = 0.755
Osteoarthritis							
Yes	14225	2792(19.1%)	1.19(1.03–1.37)^†^	1.13(0.97–1.32)p = 0.114	1104(7.7%)	1.13(0.91–1.40)p = 0.270	1.06(0.85–1.33)p = 0.603
No	15795	2765(17.5%)	1.11(0.95–1.30)p = 0.198	1.04(0.88–1.22)p = 0.647	1138(7.2%)	1.36(1.08–1.71)^†^	1.23(0.96–1.57)p = 0.101
Osteoporosis							
Yes	3701	815(22.0%)	1.00(0.76–1.32)p = 0.985	0.94(0.71–1.25)p = 0.683	1926(7.3%)	0.95(0.62–1.45)p = 0.817	0.96(0.62–1.48)p = 0.842
No	26349	4679(17.8%)	1.17(1.05–1.31)^†^	1.12(0.99–1.26)p = 0.077	316(8.5%)	1.28(1.08–1.52)^†^	1.17(0.98–1.40)p = 0.089
Rheumatoid arthritis							
Yes	736	181(24.6%)	1.54(0.87–2.75)p = 0.141	1.94(1.03–3.63)^†^	62(8.4%)	0.89(0.36–2.18)p = 0.792	0.76(0.29–1.99)p = 0.571
No	29314	5313(18.1%)	1.15(1.04–1.28)^†^	1.07(0.95–1.19)p = 0.265	2180(7.4%)	1.28(1.10–1.49)^†^	1.15(0.97–1.36)p = 0.106
Depression							
Yes	2584	1101(42.6%)	0.76(0.58–0.97)^†^	0.75(0.56–0.99)^†^	533(20.6%)	0.86(0.60–1.23)p = 0.397	0.87(0.59–1.28)p = 0.478
No	27466	4393(16.0%)	1.23(1.10–1.37)^‡^	1.17(1.03–1.31)^†^	1709(6.2%)	1.36(1.14–1.61)^‡^	1.21(1.00–1.45)^†^

OR, odds ratio; CI, confidence interval.

Adjusted for hypertension, osteoarthritis, osteoporosis, rheumatoid arthritis and depression except for the variable of own strata.

^†^p < 0.05 means “indicates significance for the comparison between patients who were and were not using drugs”.

^‡^p < 0.001 means “indicates significance for the comparison between patients who were and were not using drugs”.

Table 4 shows the ORs for developing fractures according to different BDZ usage by elderly patients. Several common BDZ subgroups including alprazolam, bromazepam, diazepam, fludiazepam, flunitrazepam, lorazepam, midazolam and other BDZs were analyzed individually. Only diazepam revealed a crude OR of 1.82 (CI: 1.32–2.50, *p* < 0.001), 1.80 (CI: 1.31–2.48, *p* < 0.001) after adjusting for use of antidepressants, antipsychotics and diuretics; and 1.49 (CI: 1.05–2.11, *p* < 0.05) after adjusting for comorbidities including hypertension, osteoarthritis, osteoporosis, rheumatoid arthritis, and depression. Use of other BDZs showed no risk after adjusting for medications or comorbidities.

**Table 4:** Risk of fractures from subgroup of benzodiazepines use in elderly insomnia patients.

Variable	Crude	Adjusted	Adjusted
	OR(95%CI)	OR*(95%CI)	OR**(95%CI)
**Benzodiazepines**	1.17(1.06–1.30)^†^	1.14(1.03–1.27)^†^	1.09(0.97–1.22)
Alprazolam	1.08(0.89–1.31)	1.05(0.86–1.28)	1.01(0.82–1.25)
Bromazepam	1.08(0.75–1.54)	1.04(0.73–1.49)	1.00(0.68–1.47)
Diazepam	1.82(1.32–2.50)^‡^	1.80(1.31–2.48)^‡^	1.49(1.05–2.11)^†^
Estazolam	1.27(1.00–1.62)^†^	1.23(0.96–1.56)	1.28(0.98–1.67)
Fludiazepam	1.04(0.80–1.33)	1.02(0.79–1.32)	1.04(0.79–1.39)
Flunitrazepam	1.00(0.59–1.70)	0.95(0.56–1.63)	1.03(0.58–1.81)
Lorazepam	0.88(0.72–1.06)	1.11(0.92–1.35)	1.05(0.86–1.28)
Midazolam	1.26(0.57–2.77)	1.20(0.54–2.64)	1.01(0.42–2.45)
Other BDZ	1.22(1.01–1.49)^†^	1.17(0.97–1.43)	1.07(0.86–1.32)

*Adjusted for the use of antidepressants, antipsychotics and diuretics.

**Adjusted for the presence of hypertension, osteoarthritis, osteoporosis, rheumatoid arthritis, and depression.

^†^p < 0.05 means “indicates significance for the comparison between patients who were and were not using drugs”.

^‡^p < 0.001 means “indicates significance for the comparison between patients who were and were not using drugs”.

Table 5 shows the ORs of different fracture sites in elderly patients using zolpidem or BDZ. Humerus, forearm and wrist sites were not associated with increased fracture risk, while hip and spine showed particularly increased risk of fractures. For hip fractures, zolpidem was associated with a crude OR of 1.53 (CI: 1.04–2.25, *p* < 0.05) and, when adjusted for medications, an OR of 1.49 (CI: 1.00–2.22, *p* < 0.05). For spine fractures, zolpidem was associated with a crude OR of 1.38 (CI: 1.07–1.78, *p* < 0.05) and, when adjusted for medications, an OR of 1.36 (CI: 1.05–1.76, *p* < 0.05). BDZ was associated with a crude OR of 1.22 (CI: 1.03–1.44, *p* < 0.05) and, when adjusted for medications, an OR of 1.20 (CI: 1.01–1.43, *p* < 0.05). However, neither site was related to increased risk of fractures after adjusting for comorbidities.

**Table 5:** Risk of different fractures at different locations due to the use of hypnotics in elderly insomnia patients.

Variable	Crude	Adjusted	Adjusted
	OR(95%CI)	OR*(95%CI)	OR**(95%CI)
**HIP**			
Benzodiazepines	1.10(0.85–1.42)	1.06(0.81–1.40)	0.99(0.75–1.30)
Zolpidem	1.53(1.04–2.25)^†^	1.49(1.00–2.22)^†^	1.25(0.83–1.87)
**Humerus**			
Benzodiazepines	1.47(0.95–2.27)	1.37(0.87–2.16)	1.43(0.90–2.29)
Zolpidem	1.28(0.67–2.44)	1.18(0.61–2.29)	1.20(0.59–2.44)
**Forearm**			
Benzodiazepines	1.20(0.88–1.62)	1.19(0.87–1.62)	1.19(0.87–1.65)
Zolpidem	1.09(0.70–1.70)	1.08(0.69–1.70)	1.05(0.66–1.69)
**Wrist**			
Benzodiazepines	1.61(0.51–5.05)	1.49(0.44–5.03)	1.64(0.53–5.12)
Zolpidem	2.00(0.18–22.05)	2.00(0.18–22.05)	4.00(0.25–63.92)
**Spine**			
Benzodiazepines	1.22(1.03–1.44)^†^	1.20(1.01–1.43)^†^	1.11(0.92–1.33)
Zolpidem	1.38(1.07–1.78)^†^	1.36(1.05–1.76)^†^	1.23(0.85–1.49)
**Other**			
Benzodiazepines	1.08(0.90–1.29)	1.14(1.03–1.27)^†^	1.09(0.97–1.22)
Zolpidem	1.13(0.87–2.46)	1.25(1.07–1.45)^†^	1.13(0.96–1.34)

*Adjusted for the use of antidepressants, antipsychotics and diuretics

**Adjusted for the presence of hypertension, osteoarthritis, osteoporosis, rheumatoid arthritis, depression.

^†^p < 0.05 means “indicates significance for the comparison between patients who were and were not using drugs”.

## Discussion

A case-crossover design was used to account for acute drug effects in patients with continued drug use over the study period. In the case-crossover design, the case has its own control, thus limiting bias resulting from gender and age, along with other genetic and physiological factors. Furthermore, it shows the immediate effects within 24 hours, rather than exposure-to-fall time of up to 6 months as in some other studies [17, 18]. Zolpidem achieves its peak plasma level within 1.6 hours of ingestion, and the single dose (5–10 mg) elimination half-life is 2.5–2.6 hours [39]. Therefore, the case-crossover approach is suitable to account for acute drug effects on fracture risk, and has been used previously to study zolpidem use and the risk of hospitalizations resulting from motor vehicle accidents [40] or fractures [18, 19, 21].

Several prior case control or case cross-over studies have found that BDZ [22, 23] and zolpidem [18, 19, 21] are associated with increased risk of fractures in the elderly. A Korean case cross-over design study found that zolpidem is associated with increased fractures among the elderly with an adjusted OR = 1.72 (95% CI, 1.37–2.16), but the dataset was taken from a shorter period (one year) and a smaller population (1508 participants) [19]. Another case cross-over study focused on a mixed nursing home population with younger patients (>50 years- old) with a 1-year follow up [21]. The present study follows a larger population (N = 6010) and a longer observation period (9 years, 2002–2010) in a nationwide cohort, and finds that zolpidem use is not associated with risk of all-site fractures after adjusting for medication use and comorbidities. The subject group is limited to elderly patients without prior history of fractures or stroke, and we adjusted for comorbidities including osteoporosis, hypertension, osteoarthritis and diabetes mellitus in addition to drug use including anti-depressants, antipsychotics and diuretics. We found that neither zolpidem nor BDZ is associated with significantly elevated fracture risk in elderly individuals with insomnia.

Some previous studies of hypnotics use in elderly patients with insomnia, found an elevated fracture risk among female patients over 60 years-old [41]. However, the present study found no such increased risk after adjusting for comorbidities and drug use.

One study reported that zolpidem use was associated with a greater risk of fractures among elderly patients than alprazolam and lorazepam us, but a similar risk to that associated with diazepam use [17]. Other studies also found that zolpidem was associated with a higher risk of fractures in the elderly [18, 42–44]. Our study, however, found that use of BDZ (18.0%) was associated with a greater risk than used of zolpidem (7.3%) in the controlled periods.

Several previous studies focused on the impact of hypnotics use on the risk of hip fractures [20, 21, 45] or nonvertebral fractures [17]. One previous study found that in elderly users of hypnotics, most fractures occurred in the femur [19]. The present study examined a range of potential fracture sites including hips, humerus, forearms, wrists, and spine. Similar to other studies, the crude ORs and ORs adjusted for medication use showed a particularly heightened risk of fractures in the hip and spine. For hip fractures, zolpidem was associated with increased fracture risk even after adjusting for other medication use. For spine fractures, both zolpidem and BDZ were associated with increased risk after adjustment. However, after adjusting for comorbidities, neither zolpidem nor BDZ was found to be associated with significantly increased risk of fractures in any particular sites. In addition, hypnotics were not found to be associated with increased risk of all-site fractures.

We also compared use of several of the BDZs most frequently prescribed to elderly people including alprazolam, bromazepam, diazepam, estazolam, fludiazepam, flunitrazepam, lorazepam and midazolam [17, 46, 47]. We found that only diazepam shows a higher OR with increased risk of fractures at 1.49 (CI = 1.05–2.11, *p* < 0.05). A possible explanation for this elevated fracture risk is that the elimination half-life of diazepam is prolonged in the elderly [48], suggesting that long half-life BDZs such as diazepam should not be prescribed to elderly patients. Other BDZs are not associate with increased of fractures after adjusting for medication use or comorbidities.

Previous studies found that depression is a risk factor associated with fractures in elderly individuals with osteoporosis [49, 50]. However, the present study found that depression actually reduces fracture risk. In our stratified analysis data, in both the BDZ and zolpidem groups, patients without depression had a higher OR than those with depression. One possible explanation for this paradoxical finding is that patients with depression are less active due to psychomotor retardation and lower energy levels [51], thus reducing the risk of fall-related fractures. However, further study might be necessary to clarify the association between depression and fracture risk.

Sleep disorders have been found to increase the risk of depression [52, 53], suicidal thoughts and behaviors [54, 55], mental symptoms in female violence victims [56], cancers [57], poor cardiorespiratory fitness [58], and increased inflammatory responses to acute emotional or cognitive stress [59]. One recent study even found that persistent insomnia may increase the risk of mortality [60]. Several studies have found that insomnia itself is an independent risk factor for falls and fractures among the elderly [61–64]. Therefore, a careful balance between zolpidem and other hypnotics is important to maintain both the mental and physical health of elderly patients with sleep disorders.

## Limitations

Patients with missing data in the LHID 2005 dataset were excluded from consideration, which could introduce minor biases in the analysis. Additionally, this study used claimed data, and we could not observe clinical events such as the extent of symptom, recovery time, actual BDZ and zolpidem dosages or side effects. In addition, before 2000 NHIRD data were coded using A-codes, which cannot be analyzed. For the ICD codes in diagnoses, there must be some individual differences, however, as aforementioned studies have shown, the NHIRD is accurate in diagnoses. Despite these limitations, the LHID provides large samples and sufficient information about disease diagnoses and drug usage, and LHID data have been used for many studies of interactions between drugs and disease or between disease and disease.

## Conclusion

This case cross-over study revealed that zolpidem or BDZ use is not associated with increased risk of all-site fractures in elderly, but that diazepam use is associated with elevated fracture risk. Increased fracture risk among patients without depression requires further investigation. Hip and spine fractures were a particular concern for elderly users of zolpidem or BDZ hypnotics, although the association was found not to be significant after adjusting for comorbidities.
